# Proof-of-Concept Evaluation of Primary Human FAP-CAR-NK Cells Targeting Activated Fibroblasts in Pulmonary Fibrosis

**DOI:** 10.3390/ijms27094128

**Published:** 2026-05-05

**Authors:** Geping Wu, Zhiming Ling, Wei Lin, Yi Wang, Xiuying Liu, Jianxun Wang

**Affiliations:** School of Life Sciences, Beijing University of Chinese Medicine, Beijing 100013, China

**Keywords:** fibroblast activation protein, CAR-NK cell, pulmonary fibrosis, immunotherapy, lung organoids

## Abstract

In recent years, the feasibility of immunotherapy targeting activated fibroblasts in pulmonary fibrosis has received further support. Recent studies have shown that transient FAP-targeted immunotherapy can alleviate pulmonary fibrosis by eliminating excessively activated fibroblasts, improving the aberrant extracellular matrix environment, and promoting alveolar cell lineage remodeling, suggesting that FAP-associated pathological stromal cells are amenable to therapeutic intervention. Based on this, research on FAP-centered engineered cell therapies is being gradually extended from settings such as myocardial fibrosis to pulmonary fibrosis. In this context, primary human NK cells represent a promising effector cell platform, as they are generally associated with a lower risk of severe treatment-related toxicities and relatively limited in vivo persistence, which may confer a more controllable therapeutic window. This feature is particularly important in fibrotic diseases, because long-term and continuous depletion of fibroblast populations may disrupt tissue homeostasis and injury repair. In addition, current studies of FAP-targeted CAR-NK therapy have mainly relied on NK cell lines such as NK-92, but these systems may not fully reflect the functional characteristics, receptor signaling, or clinical potential of primary human NK cells. Based on these considerations, it is necessary to develop a FAP-targeted cell therapy platform with greater clinical relevance for pulmonary fibrosis. In this study, we established a primary human FAP-CAR-NK-cell platform and conducted a proof-of-concept evaluation in pulmonary fibrosis-related models, including in vitro systems, a human pulmonary fibrosis-like organoid model, and an acute in vivo observation model. The main novelty of this study lies in the use of primary human NK cells for FAP-targeted intervention in pulmonary fibrosis-related models. We focused on whether these engineered cells could selectively target and eliminate FAP-positive activated fibroblasts, retain effector function in a fibrotic microenvironment, and show short-term feasibility after adoptive transfer. The study was not intended to assess long-term therapeutic efficacy or systemic safety, but rather to examine the feasibility of FAP-directed fibroblast targeting by primary human CAR-NK cells in pulmonary fibrosis and to provide a basis for further preclinical investigation.

## 1. Introduction

Fibrotic lung diseases such as idiopathic pulmonary fibrosis (IPF) still lack effective treatments capable of reversing established fibrotic architecture. Although existing antifibrotic drugs such as pirfenidone and nintedanib can slow disease progression to some extent, they are unable to eliminate established pathological stromal remodeling [1,2]. Recent breakthroughs in disease mechanisms have highlighted aberrantly activated stromal cells and their remodeled extracellular matrix (ECM) as pivotal drivers of pulmonary fibrosis. Consequently, therapeutic strategies centered on the selective depletion or precision targeting of these cell populations have gained significant momentum [3,4,5].

Fibroblast activation protein (FAP) is a type II transmembrane serine protease enriched in activated stromal compartments in malignancy, inflammation, tissue repair, and fibrotic diseases, while showing lower expression in most resting adult tissues [6]. In interstitial lung diseases, FAP expression has been reported to increase in fibrotic lung lesions [7], and 68Ga-FAPI-04 PET-CT has been used as a molecular imaging approach to assess fibroblast activation and risk in systemic sclerosis-associated interstitial lung disease [8]. Recent advances in spatial omics studies have provided more direct pathological support for this strategy [9]. In a 2025 spatial transcriptomics study, Vannan et al. reported marked spatial heterogeneity in pulmonary fibrotic lesions [10], with multiple distinct pathological programs coexisting across different local niches. Pathological regions associated with epithelial remodeling were enriched for activated fibrotic fibroblasts expressing CTHRC1, FAP, and POSTN, suggesting that FAP may be better understood as a spatially specific, disease state-dependent marker of pathological stroma rather than simply a general fibroblast marker. Subsequently, Yan et al. reported in 2025 that FAP-targeted CAR-T cells generated through in vivo LNP-mRNA delivery were able to eliminate excessively activated fibroblasts, improve the abnormal ECM environment, and promote alveolar epithelial cell lineage remodeling, thereby exerting antifibrotic effects in a mouse model of pulmonary fibrosis [11]. Collectively, these findings establish a robust proof-of-concept foundation for the targeted immune-mediated depletion of FAP-positive pathological stromal cells in pulmonary fibrosis.

Despite this progress, important gaps remain in current studies of FAP-targeted cell therapy. In pulmonary fibrosis, current proof-of-concept evidence has been derived mainly from CAR-T platforms, whereas studies of FAP-targeted CAR-NK therapy have largely relied on immortalized cell lines such as NK-92. For example, a 2025 study showed that FAP-CAR-NK-92 cells could reduce cardiac fibrosis; however, NK-92 cells may not fully reflect the receptor profile or microenvironmental responsiveness of primary human NK cells [12,13]. Therefore, it remains necessary to evaluate FAP-targeted primary human CAR-NK cells in pulmonary fibrosis models with greater clinical relevance.

Primary human NK cells may offer a distinct effector-cell platform for this indication. Early clinical experience with cord blood-derived CD19 CAR-NK cells showed antitumor activity without cytokine release syndrome, neurotoxicity, or graft-versus-host disease in that cohort, supporting the broader clinical feasibility of CAR-NK approaches [14]. Recent preclinical work has also shown that engineered NK-cell platforms can be adapted through logic-gated circuits or transient mRNA-LNP engineering, although effector source and manufacturing strategy strongly influence function [15,16]. For fibrotic diseases, the relatively limited in vivo persistence of NK cells may be advantageous because continuous depletion of FAP-positive stromal cells could disrupt physiological tissue repair or nonpulmonary stromal compartments. Consistent with this concern, systemic depletion of FAP-expressing stromal cells caused cachexia and anemia in preclinical models, and fibroblast populations also participate in wound healing and tissue homeostasis [17,18]. Safety studies of cross-reactive FAP-CAR constructs further emphasize the need to define an appropriate therapeutic window before clinical translation [19]. Human three-dimensional organoid systems provide an additional bridge between two-dimensional cytotoxicity assays and in vivo models. Distal lung organoid platforms have been developed for interstitial lung disease modeling [20], fibrotic alveolar transitional states can be modeled using pluripotent stem-cell-derived alveolar organoids [21], and recent work has extended organoids toward immune-organ interaction models, including systems containing autologous tissue-resident immune compartments [22,23].

In this study, we established a second-generation FAP-targeted CAR-NK cell platform derived from human PBMCs and evaluated its activity in pulmonary fibrosis-related models. Using in vitro assays, a TGF-β-induced activated fibroblast model, a human pulmonary fibrosis organoid co-culture system, and an acute murine model, we examined whether these engineered cells could selectively recognize and eliminate FAP-positive fibroblasts and retain functional activity in fibrosis-related microenvironments, thereby providing proof-of-concept support for further preclinical investigation of this strategy.

## 2. Results

### 2.1. Construction and Preliminary Functional Validation of PBMC-Derived FAP-CAR-NK Cells

**Figure 1:**
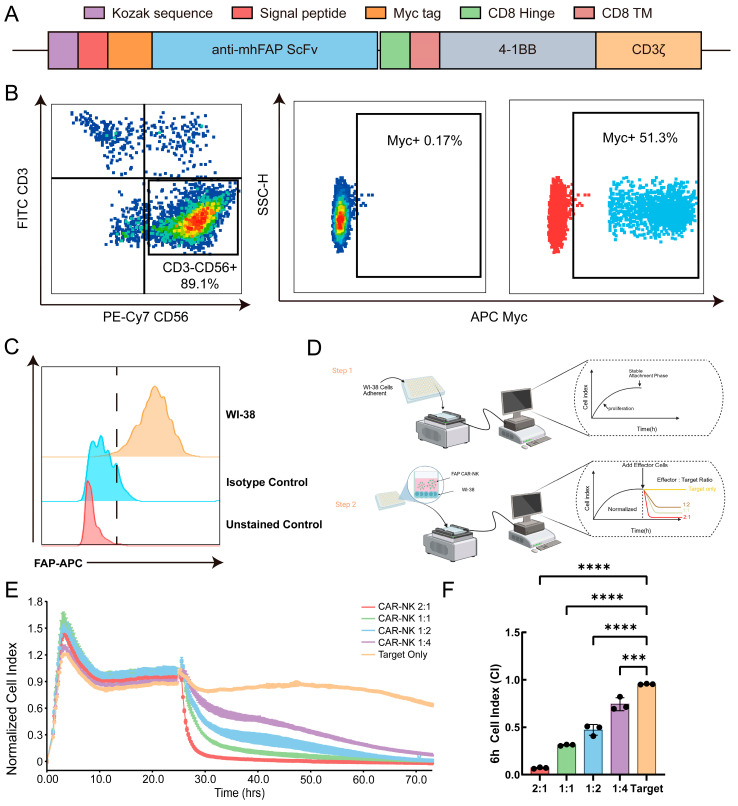
Construction and baseline functional validation of PBMC-derived FAP-CAR-NK cells. (**A**) Schematic of the second-generation FAP-CAR comprising a CD8α signal peptide, Myc tag, anti-FAP scFv, CD8α hinge and transmembrane domains, a 4-1BB costimulatory domain, and a CD3ζ signaling domain. (**B**) Representative flow-cytometric analysis of NK-cell purity and CAR surface expression after retroviral transduction, with CAR detected by Myc staining within the CD3−CD56+ gate. (**C**) Flow-cytometric validation of endogenous FAP expression in WI-38 fibroblasts. (**D**) Schematic workflow of the xCELLigence real-time cell analysis (RTCA) assay used to assess cytotoxicity against adherent WI-38 target cells. (**E**) Representative normalized cell index (CI) curves of WI-38 fibroblasts co-cultured with FAP-CAR-NK cells at the indicated E:T ratio; target-only wells served as controls. (**F**) Quantification of CI at 6 h after effector-cell addition. Data are presented as mean ± SEM from three independent donor-derived experiments (*n* = 3). *** *p* < 0.001, **** *p* < 0.0001.

We generated a second-generation FAP-targeted CAR comprising a CD8α signal peptide, Myc tag, M036 scFv, CD8α hinge and transmembrane domains, a 4-1BB costimulatory domain, and a CD3ζ signaling domain (Figure 1A). Transfection of Phoenix-Ampho packaging cells with the FAP-CAR plasmid yielded a transfection efficiency of 78.6%, and subsequent transduction of BaEV packaging cells achieved an efficiency of 91.6% (Supplementary Figure S1A). Following retroviral transduction of expanded PBMC-derived NK-cell cultures, flow-cytometric analysis of the CD3^−^CD56^+^ population showed an NK-cell purity of 89.1%, with CAR expression detected in 51.3% of gated cells (Figure 1B).

To evaluate the suitability of WI-38 fibroblasts as endogenous target cells in this system, FAP expression in WI-38 cells was first verified by flow cytometry (Figure 1C). We then evaluated the cytolytic activity of FAP-CAR-NK cells against WI-38 cells using the xCELLigence real-time cell analysis (RTCA) platform, and the experimental workflow is illustrated in Figure 1D. WI-38 cells were seeded to form confluent monolayers, after which FAP-CAR-NK cells were added at different E:T ratios (2:1, 1:1, 1:2, and 1:4), with target-only wells included as controls. As shown in Figure 1E, FAP-CAR-NK cells induced an E:T ratio-dependent decline in cell index (CI). At higher E:T ratios, CI decreased rapidly within approximately 6 h after effector-cell addition, whereas lower E:T ratio produced a slower and less complete decline, while remaining below the target-only control condition. Further quantitative analysis of CI values at the 6 h co-culture time point showed that, compared with the control group, FAP-CAR-NK cells exhibited significant cytotoxic activity at all tested effector-to-target ratios, and a clear killing effect was still observed at the low ratio of 1:4 (Figure 1F). These results indicate that WI-38 cells can serve as endogenous FAP-positive target cells for preliminary validation of CAR-mediated cytotoxicity even under relatively low effector cell conditions.

### 2.2. Antigen-Dependent Effector Activation of FAP-CAR-NK Cells Against Engineered FAP-Positive Target Cells

**Figure 2:**
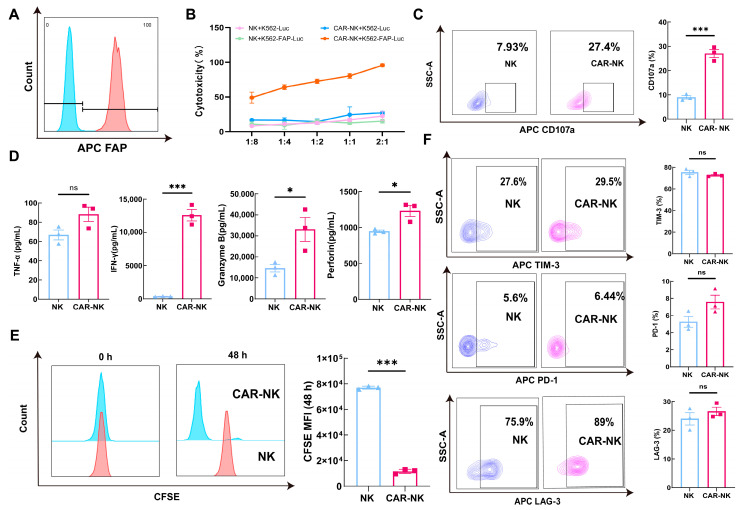
Antigen-dependent effector activation of FAP-CAR-NK cells against engineered FAP-positive target cells. (**A**) Flow-cytometric validation of surface FAP expression in engineered K562-FAP-Luc cells. (**B**) Luciferase-based cytotoxicity assay comparing untransduced NK cells and FAP-CAR-NK cells against K562-Luc and K562-FAP-Luc target cells at the indicated E:T ratio after 12 h co-culture. (**C**) Representative flow-cytometric analysis and quantification of CD107a surface expression in untransduced NK cells and FAP-CAR-NK cells after stimulation with K562-FAP-Luc target cells, after 4 h co-culture. (**D**) Multiplex CBA quantification of TNF-α, IFN-γ, granzyme B, and perforin in co-culture supernatants after 4 h stimulation with K562-FAP-Luc target cells. (**E**) CFSE-based proliferation analysis of untransduced NK cells and FAP-CAR-NK cells after 48 h co-culture with K562-FAP-Luc target cells. (**F**) Representative flow-cytometric analysis and quantification of TIM-3, PD-1, and LAG-3 expression in untransduced NK cells and FAP-CAR-NK cells after co-culture with K562-FAP-Luc target cells. Data are presented as mean ± SEM from three independent donor-derived experiments (*n* = 3). * *p* < 0.05, *** *p* < 0.001, ns, not significant.

To further evaluate antigen-dependent responses in a defined target system, we generated K562-FAP-Luc reporter cells expressing human FAP and luciferase, and the corresponding plasmid map is shown in Supplementary Figure S2A. Surface FAP expression in the engineered cells was confirmed by flow cytometry (Figure 2A). Luciferase-based cytotoxicity assays were then performed to compare the killing activity of untransduced NK cells and FAP-CAR-NK cells against K562-Luc and K562-FAP-Luc target cells. As shown in Figure 2B, untransduced NK cells and FAP-CAR-NK cells displayed comparable cytotoxic activity against K562-Luc targets, whereas FAP-CAR-NK cells exhibited enhanced cytotoxicity against K562-FAP-Luc targets across the tested E:T ratios, indicating antigen-dependent augmentation of killing in this engineered target system.

To further determine whether antigen recognition was accompanied by enhanced effector cell activation, we next assessed CD107a surface expression by flow cytometry. Following stimulation with K562-FAP-Luc target cells, FAP-CAR-NK cells showed increased CD107a expression compared with untransduced NK cells, consistent with enhanced degranulation upon recognition of FAP-positive targets (Figure 2C). Cytokine secretion in the supernatant was further measured at an E:T ratio of 1:2. Supernatant cytokine analysis showed that, following co-culture with K562-FAP-Luc target cells, FAP-CAR-NK cells secreted higher levels of effector-associated cytokines than untransduced NK cells, indicating enhanced effector activation in response to FAP-positive target stimulation. TNF-α showed no significant difference, likely because its release kinetics fluctuate substantially within a short co-culture window. The current data therefore suggest that the main advantage conferred by CAR expression is more closely associated with perforin/granzyme and IFN-γ-related effector functions (Figure 2D).

CFSE analysis further demonstrated a greater reduction in CFSE mean fluorescence intensity (MFI) in FAP-CAR-NK cells than in untransduced NK cells after co-culture with FAP-positive targets, supporting enhanced proliferative activity following antigen exposure (Figure 2E). In addition, exploratory analysis of the exhaustion-associated markers PD-1, LAG-3, and TIM-3 did not reveal significant differences between groups under these short-term co-culture conditions (Figure 2F). Taken together, these data indicate that recognition of engineered FAP-positive targets is accompanied by enhanced killing, degranulation, cytokine secretion, and proliferative responses in FAP-CAR-NK cells, without detectable early upregulation of the examined exhaustion-associated markers after a single round of stimulation.

### 2.3. Functional Evaluation of FAP-CAR-NK Cells in a TGF-β-Induced MRC-5 Activation Model

**Figure 3:**
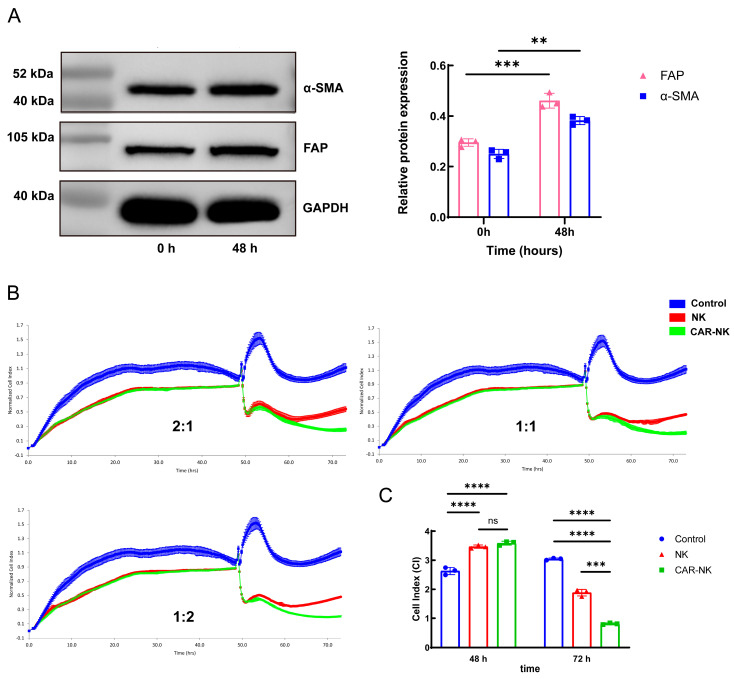
Functional evaluation of FAP-CAR-NK cells in a TGF-β-induced activated fibroblast model. (**A**) Western blot analysis and densitometric quantification of α-SMA and FAP expression in MRC-5 cells before and after 48 h of stimulation with 5 ng/mL TGF-β; GAPDH served as the loading control. (**B**) Representative RTCA normalized CI curves of TGF-β-induced MRC-5 cells co-cultured with untransduced NK cells or FAP-CAR-NK cells at E:T ratio of 2:1, 1:1, and 1:2; target cell-only wells served as controls. (**C**) Quantification of CI at the time of effector-cell addition (48 h) and at the 24 h co-culture endpoint (72 h). Statistical comparisons at 48 h reflect differences between untreated and TGF-β-stimulated target-cell conditions before effector-cell addition and were not used to infer effector-cell cytotoxicity. Data are presented as mean ± SEM from three independent donor-derived experiments (*n* = 3). ** *p* < 0.01, *** *p* < 0.001, **** *p* < 0.0001, ns, not significant.

To establish a disease-related fibroblast target model that more closely reflects the pathological state of pulmonary fibrosis, MRC-5 cells were first serum-starved in medium containing 1% FBS for 6 h, followed by stimulation with 5 ng/mL TGF-β for 48 h. To verify the induction effect, cells were collected at 0 h and 48 h for Western blot analysis. As shown in Figure 3A, α-SMA and FAP expression increased after 48 h of TGF-β stimulation, consistent with the acquisition of an activated fibroblast-like phenotype.

Based on these results, TGF-β-induced MRC-5 cells at 48 h were selected as disease-related target cells for subsequent cytotoxicity assays. After 48 h of induction, untransduced NK cells or FAP-CAR-NK cells were added at E:T ratio of 2:1, 1:1, and 1:2, and the killing process was continuously monitored by RTCA for 24 h (Figure 3B). The results show that, at all tested E:T ratios, both NK cells and FAP-CAR-NK cells exerted cytotoxic effects on the target cells, but the killing activity of FAP-CAR-NK cells was consistently stronger than that of untransduced NK cells. Further analysis of the normalized CI curves showed that, at the endpoint, the NK group exhibited a certain degree of CI recovery, suggesting that its cytotoxic effect against target cells weakened over time. In contrast, the curve of the FAP-CAR-NK group remained relatively stable and suppressed, indicating a more sustained inhibitory effect on the target cells.

Consistent with these findings, endpoint CI analysis showed that both the NK group and the FAP-CAR-NK group significantly reduced CI values compared with the control group, and the magnitude of CI reduction was greater in the FAP-CAR-NK group than in the NK group at all tested E:T ratios (Figure 3C). These results indicate that, in the TGF-β-induced activated MRC-5 model, FAP-CAR-NK cells exhibited stronger and more sustained cytotoxic activity than untransduced NK cells. Because TGF-β stimulation itself increased the CI of MRC-5 cells before effector-cell addition, the CI values at 48 h reflected the baseline difference between untreated control cells and TGF-β-activated target cells, rather than any effect of NK or FAP-CAR-NK cells. Therefore, the cytotoxic effect was interpreted primarily from the post-effector-addition kinetics and endpoint CI changes after co-culture.

### 2.4. Proof-of-Concept Activity of FAP-CAR-NK Cells in a Human Pulmonary Fibrosis Organoid Co-Culture Model

**Figure 4:**
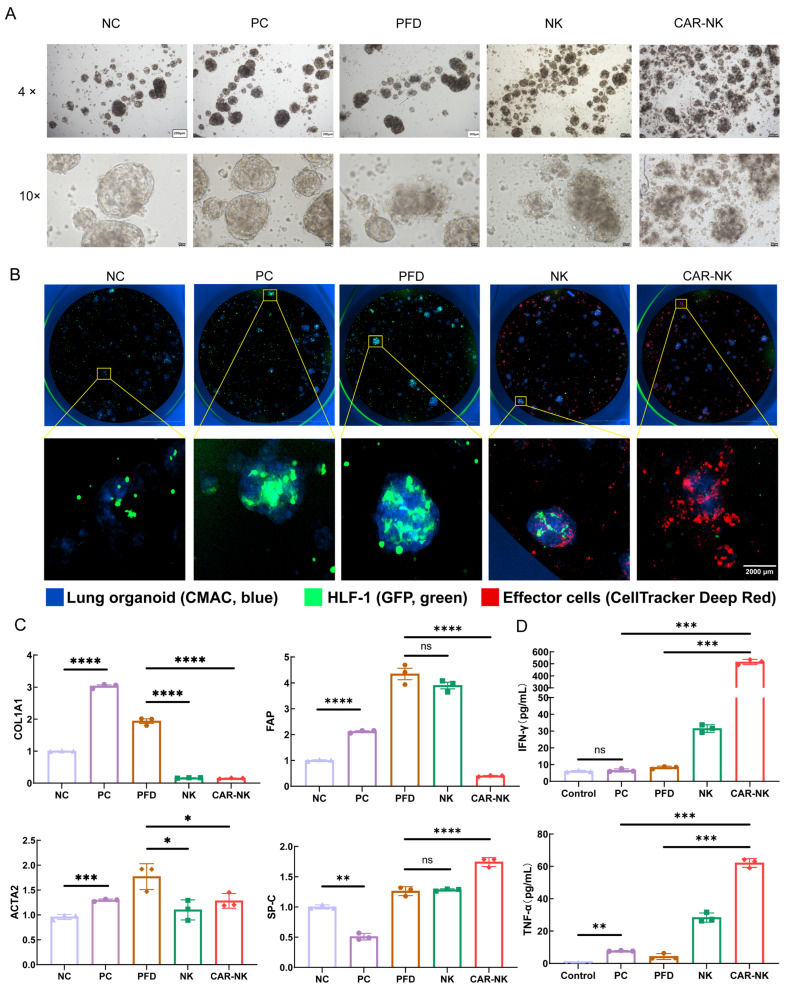
Proof-of-concept activity of FAP-CAR-NK cells in a fibrotic human lung organoid co-culture model. (**A**) Bright-field images showing morphological alterations in lung organoid co-cultures under non-fibrotic control conditions (NC), profibrotic stimulation (PC), pirfenidone treatment (PFD), untransduced NK-cell treatment (NK), and FAP-CAR-NK-cell treatment (CAR-NK). Treatment-associated fragmented, debris-like material is particularly evident in the NK and CAR-NK groups. Images were acquired at 4× and 10× magnification. (**B**) Multichannel fluorescence imaging of the three-dimensional Matrigel microdroplet co-culture system showing lung organoids labeled with CellTracker Blue CMAC (blue), HFL-1 fibroblasts labeled with GFP (green), and effector cells labeled with CellTracker Deep Red (red); lower panels show magnified views of representative droplets (24 h). (**C**) RT-qPCR analysis of COL1A1, FAP, ACTA2, and SP-C expression in the indicated groups. qPCR primers are shown in Supplementary Table S2. (**D**) Quantification of IFN-γ and TNF-α in culture supernatants after co-culture. Data are presented as mean ± SEM from three independently established organoid co-culture experiments (*n* = 3). * *p* < 0.05, ** *p* < 0.01, *** *p* < 0.001, **** *p* < 0.0001, ns, not significant.

To evaluate FAP-CAR-NK activity in a three-dimensional human tissue environment, we established a fibrotic lung organoid co-culture model by combining human pulmonary organoids with hypoxia-conditioned FAP-positive fibroblasts using a droplet-based microfluidic encapsulation system. Following pro-fibrotic stimulation with TGF-β and LPS, the model group exhibited disrupted spherical morphology and formation of a fibroblast-rich peripheral layer surrounding epithelial-like structures, giving rise to a typical core-shell-like pattern (Figure 4A). After treatment with pirfenidone, untransduced NK cells, or FAP-CAR-NK cells, these abnormal structures showed varying degrees of improvement. Notably, at low magnification, the NC, PC, and pirfenidone groups showed relatively clean backgrounds, whereas the NK and FAP-CAR-NK groups contained more debris-like and fragmented material. At higher magnification, disruption of the fibroblast-rich peripheral shell was observed, together with a reduction in fibroblast-like components and local cellular fragmentation and detachment, and these changes were most evident in the FAP-CAR-NK group. Taken together, these morphological findings suggest that effector cell treatment promoted loosening of the fibrotic encapsulating structure and support a direct effect on the fibroblast-containing outer layer.

Cytation 5 imaging showed that organoids, fibroblasts, and effector cells remained organized within the Matrigel microdroplet system during co-culture, and that NK/CAR-NK cells localized in proximity to fibroblast-containing structures (Figure 4B). Additional intermediate time-point images are shown in Supplementary Figure S3.

RT-qPCR analysis showed that, compared with the control condition, pro-fibrotic stimulation reduced SP-C expression and increased FAP and COL1A1 expression. After FAP-CAR-NK treatment, SP-C expression showed partial recovery, whereas FAP and COL1A1 expression were significantly decreased. Notably, ACTA2 did not show a pattern consistent with that of FAP and COL1A1 across groups. Given that ACTA2 more closely reflects the myofibroblast phenotype and that its expression may be influenced by the heterogeneity of the organoid co-culture system, the selected observation time point, and the distinct effects of different interventions, ACTA2 did not serve as a sensitive indicator of overall improvement at this endpoint (Figure 4C). Cytokine analysis of culture supernatants further showed increased IFN-γ and TNF-α levels in the FAP-CAR-NK group, while the other measured cytokines showed no significant differences between groups (Figure 4D).

### 2.5. Histological and Immunohistochemical Evaluation Further Supported Structural and Stromal Changes in Organoid Sections

**Figure 5:**
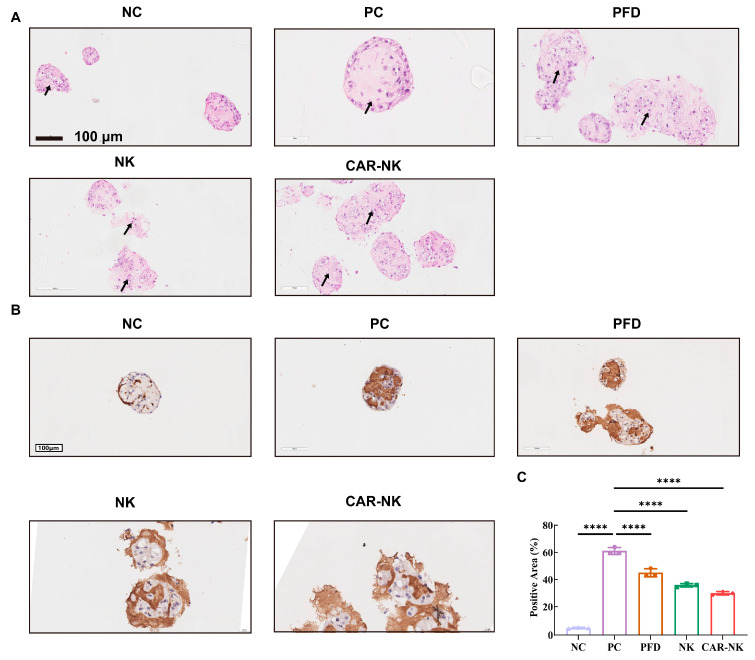
Histological and immunohistochemical evaluation of organoid sections. (**A**) Representative H&E-stained sections of organoid co-cultures from the NC, PC, PFD, NK, and CAR-NK groups. Black arrows indicate representative morphological features in each group, including loose organoid organization in the NC group, compact spheroid-like structures in the PC group, and disrupted or less compact residual organoid structures after treatment. (**B**) Representative FAP immunohistochemistry of organoid sections from the same groups. (**C**) Quantification of the FAP-positive area in organoid sections. Scale bars, 100 μm. Data are presented as mean ± SEM from three independently established organoid co-culture samples (*n* = 3). **** *p* < 0.0001, ns, not significant.

Representative H&E staining (Figure 5A) showed that organoids in the NC group were loosely arranged and lacked a well-defined spheroid architecture, whereas the PC group displayed more compact and enlarged spheroid-like structures. After treatment, spheroid integrity was disrupted to varying degrees, with looser overall organization and increased flattened gland-like structures. This pattern was more apparent in the CAR-NK group, in which the residual organoid structures appeared smaller and less compact than those in the PC group.

Representative FAP immunohistochemistry (Figure 5B) showed minimal staining in the NC group and markedly increased stromal FAP signal in the PC group. Compared with the PC group, FAP-positive area was reduced in all treatment groups, with the lowest value observed in the CAR-NK group. These findings support a reduction in FAP-associated stromal features after CAR-NK treatment; quantification of the FAP-positive area is shown in Figure 5C.

### 2.6. In Vivo Feasibility and Target Engagement of FAP-CAR-NK Cells in an Irradiated Bleomycin Lung Injury Model

**Figure 6:**
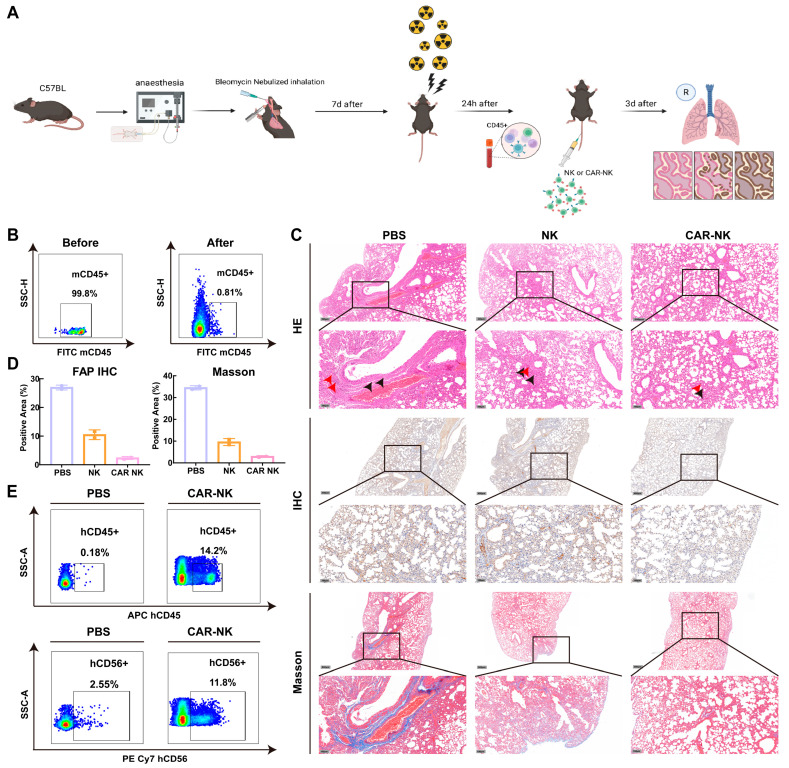
Acute in vivo feasibility and short-term target engagement of FAP-CAR-NK cells in an irradiated bleomycin lung-injury model. (**A**) Schematic of the experimental design, including bleomycin administration, fractionated total body irradiation (TBI), infusion of PBS, untransduced NK cells, or FAP-CAR-NK cells with rhIL-15 support, and acute tissue collection. (**B**) Representative flow-cytometric analysis of peripheral blood CD45+ leukocytes before TBI and 24 h after TBI. (**C**) Representative H&E staining, FAP immunohistochemistry, and Masson’s trichrome staining of lung tissues collected 72 h after infusion. (**D**) Quantitative analysis of the FAP-positive area by immunohistochemistry and the collagen-positive area by Masson’s trichrome staining in lung tissues from surviving animals at the acute endpoint. (**E**) Representative flow-cytometric assessment of acute pulmonary homing of human cells in lung tissue collected 24 h after infusion, showing hCD45+ and hCD56+ populations. Owing to the short observation window, irradiation-based preconditioning, and substantial peri-procedural mortality, these data should be interpreted as preliminary evidence of acute in vivo feasibility, lung tissue access, and short-term target engagement rather than definitive evidence of therapeutic efficacy or systemic safety.

To evaluate short-term in vivo feasibility of human effector cells under host conditions more favorable for their transient persistence, we established a bleomycin (BLM)-induced lung-injury model in C57BL/6J mice combined with fractionated total body irradiation (TBI) as host preconditioning to support the temporary persistence of infused human effector cells (Figure 6A). Flow-cytometric analysis of peripheral blood 24 h after TBI confirmed marked depletion of circulating CD45^+^ leukocytes, with the CD45^+^ fraction decreasing from 99.8% before irradiation to 0.81% after preconditioning (Figure 6B). At 72 h post-TBI, mice received tail vein infusion of PBS, untransduced NK cells, or FAP-CAR-NK cells, together with a single dose of rhIL-15, and lung tissues were subsequently collected at 72 h later for acute endpoint for analysis.

Histological analysis of lung tissues after infusion showed evident interstitial thickening and inflammatory-cell infiltration in the PBS group. In contrast, both the NK and FAP-CAR-NK groups showed directionally milder pathological changes on H&E staining, with the overall abnormalities being least pronounced in the FAP-CAR-NK group (Figure 6C). Consistent with these observations, Masson’s trichrome staining showed the highest collagen deposition in the PBS group, while quantitative image analysis demonstrated lower fibrotic burden in the NK and FAP-CAR-NK groups, with the lowest levels observed in the FAP-CAR-NK group. FAP immunohistochemistry showed the strongest stromal FAP signal in the PBS group and reduced staining in both treatment groups, again with the lowest signal in the FAP-CAR-NK group (Figure 6D). Given the limited number of animals ultimately included in this acute model, these histological and immunohistochemical findings should be regarded as preliminary evidence of acute feasibility and short-term target engagement, reflecting early changes consistent with the study hypothesis rather than definitive evidence of therapeutic efficacy or safety. The inferential value of this model is further limited by its dependence on irradiation-based preconditioning, the very short observation window, and the high peri-procedural mortality.

To examine whether infused human effector cells reached the injured lung, an additional cohort was collected 24 h after infusion for flow-cytometric assessment of pulmonary homing. In the PBS group, only a very low level of background human hCD45 signal was detected in lung tissue (0.18%). In contrast, the proportion of hCD45-positive cells increased to 14.2% in the FAP-CAR-NK group. Further analysis showed that the hCD56-positive signal was also higher in the FAP-CAR-NK group than in the PBS group (11.8% vs. 2.55%) (Figure 6E). These data indicate that infused human NK-derived effector cells can be detected in the injured lung within the acute observation window.

Peri-procedural mortality was substantial in this model, with deaths observed in all groups during the conditioning and early post-infusion period (PBS, 2/4; NK, 1/4; FAP-CAR-NK, 2/4). In the NK group, one additional animal remained alive but was in a moribund condition before the planned collection time and was therefore excluded from tissue-based analysis to avoid confounding by severe preterminal stress. Therefore, this part of the study is more appropriately interpreted as an acute proof-of-concept evaluation of in vivo feasibility, lung tissue access, and early target engagement, rather than as an assessment of long-term efficacy or systemic safety.

## 3. Discussion

The central novelty of this study does not lie in proposing a new FAP target or a new CAR design, but in applying primary human PBMC-derived NK cells to FAP-targeted intervention in pulmonary fibrosis-related models and conducting a systematic proof-of-concept evaluation within a stepwise framework consisting of an engineered target-cell system, a TGF-β-induced activated fibroblast model, a human pulmonary fibrosis-like organoid co-culture system, and an acute in vivo observation model. In particular, the introduction of a human three-dimensional organoid model allowed this study to move beyond two-dimensional killing assays and to evaluate the functional performance of FAP-CAR-NK cells under conditions that more closely resemble the tissue microenvironment, thereby bridging, to some extent, in vitro observations at the molecular level with findings from the acute in vivo feasibility study. Based on the current data, this study supports three main conclusions. First, primary human FAP-CAR-NK cells exhibit enhanced antigen-dependent cytotoxicity and effector activation in an FAP-positive setting. Second, this enhanced activity can be at least partially retained in a three-dimensional human fibrosis-like microenvironment with spatial structure and matrix constraints. Third, under host conditions that are technically favorable for the transient persistence of human cells, signals associated with infused human effector cells can be detected in lung tissue within the acute observation window. Taken together, these findings provide proof-of-concept support for primary human FAP-CAR-NK cells as a candidate cell therapy strategy for pulmonary fibrosis. However, the current evidence remains at an early preclinical stage and is not sufficient to support definitive conclusions regarding long-term efficacy or systemic safety.

At the in vitro level, this study first verified the functional gain conferred by the CAR construct in a target-cell system with a clearly defined antigen background. Consistent with previous studies of FAP-CAR-NK cells based on the NK-92 platform [12], we observed in the K562-FAP-Luc model that FAP-CAR-NK cells exhibited stronger cytotoxicity, degranulation, cytokine release, and proliferative responses than untransduced NK cells, whereas the two groups showed comparable killing activity against FAP-negative K562-Luc cells. These findings suggest that the advantage conferred by CAR modification in this study was primarily manifested as antigen-dependent enhancement against FAP-positive target cells, rather than nonspecific amplification of the basal activity of NK cells. Given that this study used PBMC-derived primary human NK cells rather than an immortalized cell line, these results also improve, to some extent, the relevance of this strategy with respect to receptor integrity and clinical translation [14,15,16]. It should be noted that mock-CAR-transduced or non-targeting vector-transduced NK cells were not included as controls in this study. Therefore, the potential effects of retroviral transduction itself or vector integration on the activation state of effector cells cannot be fully excluded. This remains a limitation that should be acknowledged in CAR-NK studies using untransduced NK cells as the control.

The cytokine findings obtained in the engineered FAP-positive target-cell system also support this interpretation, although overinterpretation should be avoided. In this study, FAP-CAR-NK cells showed stronger release of effector-related cytokines after co-culture with K562-FAP-Luc cells, whereas TNF-α did not reach statistical significance. A more cautious interpretation is that, within the short co-culture window used in this study, the release kinetics of different cytokines may not be uniform. Accordingly, the current data support the view that FAP-CAR-mediated enhancement is mainly reflected at the level of overall effector activation, but do not permit more detailed conclusions regarding specific effector pathways on the basis of a single cytokine result. Rather than interpreting the non-significant difference in TNF-α as contradictory to the overall conclusion, it is more appropriate to regard it as a local inconsistency under the present observation window and assay conditions, which will require further clarification in more systematic functional studies.

This antigen-dependent enhancement was further supported in the TGF-β-induced activated MRC-5 model. In this model, upregulated expression of FAP and α-SMA in target cells indicated acquisition of an activated fibroblast-like phenotype more closely related to the disease state [24,25,26]. In this setting, both untransduced NK cells and FAP-CAR-NK cells showed inhibitory effects, but the latter exerted stronger and more sustained inhibition. These findings indicate that the advantage conferred by CAR expression was not restricted to an engineered overexpression system but could also be retained in a disease-related activated fibroblast model. Caution is warranted, however, in that the baseline inhibitory activity of untransduced NK cells is more appropriately interpreted as being consistent with the intrinsic antifibrotic activity of NK cells reported previously, while the underlying mechanisms were not directly investigated in this study. Therefore, it would not be appropriate at this stage to attribute this effect to specific IFN-γ-mediated or other signaling pathways. In other words, the present study supports the conclusion that CAR modification provides a targeted enhancement against FAP-positive activated fibroblasts on the basis of the native biology of NK cells [27].

To a certain extent, this organoid model preserves pulmonary fibrosis-related microenvironmental features, including epithelial–stromal interactions, spatial tissue architecture, and matrix constraints, and therefore provides a higher-level platform for evaluating the function of FAP-CAR-NK cells under conditions more closely related to the pathological state. In this study, FAP-CAR-NK treatment was associated with loosening and disruption of the fibroblast-rich peripheral shell-like structure, accompanied by downregulation of FAP and COL1A1 and partial restoration of SP-C. These findings suggest that FAP-CAR-NK cells were not only able to access and act on fibroblast-rich pathological regions in a three-dimensional environment, but that their activity was also associated with attenuation of fibrosis-related stromal features. This decrease was directionally consistent with the findings of Yan et al., in which elimination of excessively activated fibroblasts by FAP-targeted CAR-T cells improved the abnormal ECM environment and was accompanied by alveolar epithelial lineage remodeling [11], suggesting that targeted elimination of FAP-positive pathological stromal cells may lead to shared biological consequences across different cell therapy platforms. In the present study, the partial recovery of SP-C may likewise reflect a similar improvement in the stromal–epithelial microenvironment, like KRT8-positive or intermediate alveolar epithelial cell states [28,29,30,31]; however, because the current model cannot distinguish direct from indirect effects, this connection remains inferential rather than causally established.

The lack of a consistent change in ACTA2 relative to FAP and COL1A1 in the organoid results also requires cautious interpretation. Compared with FAP and COL1A1, which more directly reflect stromal burden and ECM-related programs, ACTA2 may correspond more closely to a specific myofibroblast-like phenotypic dimension, and its expression may be more readily influenced by the observation time point, the compositional heterogeneity of the organoid system, and the mode of intervention. Therefore, the fact that ACTA2 did not decrease in parallel at the selected endpoint does not necessarily weaken the trend toward stromal alleviation suggested by FAP and COL1A1, but may instead reflect heterogeneity within pathological stromal populations. This interpretation is also consistent with recent spatial transcriptomic studies showing stromal heterogeneity in pulmonary fibrosis, in which pro-fibrotic fibroblasts do not form a single continuous axis but instead comprise multiple spatially coupled pathological communities with only partially overlapping functions. CTHRC1^high^ myofibroblasts are not fully equivalent to the myofibroblast phenotypic dimension reflected by ACTA2, and regulation of the latter may be influenced by additional temporal and spatial factors [9,10,32,33]. Accordingly, based on the current data, a more appropriate conclusion is that different fibrosis-related markers may reflect distinct dimensions of pathological stroma. While preliminary evidence supports an effect of FAP-CAR-NK cells on some of these dimensions, the available data do not demonstrate uniform reversal of all activation programs.

To verify the intervention ability of FAP-CAR-NK cells on the pulmonary fibrosis microenvironment in vivo, this study used a bleomycin-induced lung injury model combined with fractionated total body irradiation to support the short-term in vivo retention of human effector cells [34]. The detection of human hCD45+ cells and hCD56+ signals in lung tissue shortly after infusion indicated that infused human NK-derived effector cells were detectable in injured lung tissue within the acute observation window. At the 72-h observation endpoint, histology and staining results show that the FAP-CAR-NK group exhibited an improving trend in collagen deposition and FAP-related stromal signals. This suggests that reduced FAP-associated stromal signal is consistent with short-term target engagement but does not establish therapeutic clearance of pathological stroma. Although animal models of pulmonary fibrosis help explore mechanisms, they cannot fully replicate the complete pathophysiology of human IPF [35]. Even with humanized immune system strategies, inherent limitations remain, such as long reconstitution cycles and mismatched cytokine environments [36]. Accordingly, the H&E, Masson’s trichrome, and FAP immunohistochemical findings should likewise be regarded as descriptive only. Although the observed changes were directionally consistent with the study hypothesis, with only a very small number of animals ultimately included in the tissue-based analysis (PBS, *n* = 2; NK, *n* = 2; FAP-CAR-NK, *n* = 2) and the observation time point falling within the acute injury phase of the BLM model, these images and quantitative data should not be interpreted as direct evidence supporting antifibrotic efficacy. More strictly, the current in vivo results primarily address whether this platform has a sufficient basis for further in vivo investigation, rather than whether it can ameliorate established fibrotic lesions.

This study provides preliminary proof-of-concept evidence for FAP-targeted CAR-NK cells in pulmonary fibrosis-related models, but several limitations remain. First, the animal experiment observation window was short and relied on irradiation preconditioning. The current results only support the feasibility of short-term in vivo homing and acute targeted engagement; they are insufficient to evaluate key issues such as long-term persistence, repeated dosing, the therapeutic window, effects on established fibrotic matrix, or systemic safety [34,36,37]. Second, although the organoid system is closer to the tissue microenvironment than 2D cultures, it still cannot fully reproduce the complex interactions among immune cells, epithelial cells, vascular components, and stromal heterogeneity in pulmonary fibrosis [20,21]. Third, this study did not systematically define the mode of target-cell death, nor did it assess functional durability or exhaustion dynamics after prolonged or repeated antigen stimulation. The current analysis of exhaustion-related molecules was limited to an exploratory evaluation after a single round of short-term co-culture and therefore cannot determine whether sustained antigen exposure would induce progressive upregulation of inhibitory receptors. Fourth, this study did not include a mock-CAR control and did not evaluate the distribution or potential toxicity of FAP-CAR-NK cells in extrapulmonary tissues with FAP expression. Accordingly, the potential effects related to retroviral transduction and the possibility of off-target risk cannot be fully excluded. Although FAP is enriched in pathologically activated fibroblasts, low-level expression can also be detected in tissues such as sites of normal wound repair and bone marrow stroma. The relevant safety boundaries therefore remain to be defined in more systematic preclinical studies [7,17,18,19].

Overall, this study shows that primary human FAP-CAR-NK cells can selectively act on FAP-positive activated fibroblasts across multiple pulmonary fibrosis-related models and retain a certain degree of functional activity in a three-dimensional human organoid system, thereby providing proof-of-concept support for this strategy as a candidate cell therapy approach for pulmonary fibrosis. At present, the available data support the in vitro and human three-dimensional model-based functional feasibility of primary human FAP-CAR-NK cells, as well as their suitability for further investigation in an acute in vivo observation system. Questions that remain unresolved include long-term antifibrotic efficacy, functional durability under repeated dosing, systemic safety, and off-target risk. These issues will need to be systematically evaluated in more advanced preclinical models.

## 4. Materials and Methods

### 4.1. Cell Lines

Phoenix-AMPHO and HFL-1 cells were obtained from the American Type Culture Collection (ATCC). K562 feeder cells expressing mbIL-21 and 4-1BBL were generated in-house. Luciferase-expressing K562 cells (K562-Luc) and FAP-overexpressing luciferase-expressing K562 target cells (K562-FAP-Luc) were established in our laboratory. WI-38 cells were purchased from Procell Life Science (Wuhan, China). BaEV-PackRV and MRC-5 cells were kindly provided by Cell Valley Biotech (Shenzhen, China). Phoenix-AMPHO, WI-38, BaEV-PackRV, and MRC-5 cells were maintained in DMEM (Gibco, #11995500BT) supplemented with 10% fetal bovine serum (FBS; Gibco, #A5256701). K562, K562-Luc, and K562-FAP-Luc cells were maintained in RPMI-1640 (Gibco, #11875500BT) supplemented with 10% FBS. HFL-1 cells were maintained in F-12K medium (Gibco, #21127-022) supplemented with 10% FBS. All cell cultures were maintained at 37 °C in a humidified incubator with 5% CO_2_ and were routinely confirmed to be free of mycoplasma contamination before use.

### 4.2. CAR Construct Design and Retrovirus Production

The FAP CAR cassette consisted of a CD8α signal peptide, an M036 scFv (reported in US Patent US10137202B2; described to recognize both human and murine FAP), a CD8α hinge and transmembrane region, a 4-1BB costimulatory domain, and a CD3ζ signaling domain. An N-terminal Myc epitope tag was included for surface detection by flow cytometry. The humanized scFv was synthesized (GenScript, Nanjing, China), and the remaining domains were derived from an in-house CAR template plasmid.

The FAP-CAR expression construct was generated in a retroviral backbone vector using standard molecular cloning procedures, and plasmid DNA for virus production was prepared using endotoxin-free methods. (TianGen Biotech, Beijing, China, #DP106).

Phoenix-AMPHO cells at ~70–80% confluence were transfected with FuGENE HD (Promega, Madison, WI, USA, #E2311) in Opti-MEM (Gibco, Grand Island, NY, USA, #11058021). Viral supernatants were collected at 48 and 72 h post-transfection, filtered through 0.45 μm filters, and stored at −80 °C. Phoenix-AMPHO-derived retroviral supernatant was used to transduce BaEV-PackRV cells to generate BaEV producer cells, and BaEV-pseudotyped viral supernatants from these cells were subsequently used for NK-cell transduction.

### 4.3. Isolation, Culture, and Expansion of NK Cells

Peripheral blood mononuclear cells (PBMCs) were isolated from healthy donor blood samples by Lymphoprep (STEMCELL Technologies, Vancouver, BC, Canada, #18061) density-gradient centrifugation and cultured at 1 × 10^6^ cells/mL in X-VIVO 15 medium (Lonza, Lonza, Basel, Switzerland, #02-060Q) supplemented with 10% FBS and recombinant human IL-2 (200 U/mL; SinoBiological, #GMP-11848-HNAE).

To expand NK cells within PBMC cultures, laboratory-generated K562 feeder cells expressing membrane-bound IL-21 (mbIL-21) and 4-1BBL were used. Before co-culture, feeder cells were treated with mitomycin C (MMC, ACMEC, #M94351; 10 µg/mL for 30 min) to prevent feeder-cell proliferation. On day 0, PBMCs were co-cultured with MMC-treated feeder cells at a 1:1 ratio. Recombinant human IL-2 was replenished daily to maintain a final concentration of 200 U/mL, and cultures were maintained at 37 °C with 5% CO_2_ until day 5, when viral transduction was performed.

PBMCs were obtained from three independent healthy donors. Unless otherwise specified, PBMC-derived cells from each donor were expanded and transduced separately and were included in the analysis as independent biological replicates; cells from different donors were not pooled. Unless otherwise indicated in the figure legends, n in in vitro experiments refers to the number of independent donor-derived biological replicates.

### 4.4. FAP CAR Transduction and Expression Analysis in NK Cells

Non-tissue culture-treated 12-well plates were coated overnight at 4 °C with 20 μg/mL RetroNectin (Takara, Kusatsu, Shiga, Japan, #T100A) diluted in PBS. After removal of the coating solution, wells were blocked with PBS containing 2% FBS for 30 min at room temperature and then rinsed with PBS. For virus loading, 2 mL of retroviral supernatant was added to each RetroNectin-coated well, and plates were centrifuged at 2000× *g* for 2 h at 32 °C. Immediately thereafter, 5 × 10^5^ cells from the expanded PBMC-derived cultures were added per well in X-VIVO 15 medium (supplemented with 10% FBS and IL-2 200 U/mL). The plates were then centrifuged at 2000× *g* for 1 h at 37 °C and incubated at 37 °C in 5% CO_2_. Cells were harvested 48 h after the final transduction, and CAR surface expression was assessed by flow cytometry via Myc-tag staining within the CD3^−^CD56^+^ NK-cell gate (antibody details are provided in Supplementary Table S1). When indicated, a second round of RetroNectin-assisted transduction was performed 24 h after the initial transduction using the same procedure.

### 4.5. FAP-CAR-NK Cytotoxicity Assay and Cytokine Detection

The cytotoxic activity of FAP-CAR-NK cells was evaluated using a luciferase-based assay with K562-Luc or K562-FAP-Luc target cells. Untransduced NK cells or FAP-CAR-NK cells were co-cultured with target cells in 96-well plates at the indicated E:T ratio for 12 h at 37 °C. After co-culture, Bio-Lumi™ luciferase substrate (Beyotime, Shanghai, China, #RG042M) was added at a 1:1 volume ratio to the culture medium, and luminescence intensity was measured as relative light units (RLU) using a multifunctional plate reader.

For cytokine measurement, co-culture supernatants were collected after 4 h of co-culture of untransduced NK cells or CAR-NK cells with K562-FAP-Luc target cells at an E:T ratio of 1:2. After centrifugation to remove cellular debris, the levels of TNF-α, IFN-γ, perforin, and GZMB in the supernatants were measured using the LEGENDplex™ Multiplex Cytokine Assay Kit (BioLegend, San Diego, CA, USA, Human CD8/NK Panel, #740939), and the data were analyzed using the manufacturer’s online analysis platform (version 2025-05-01).

### 4.6. Real-Time Cytotoxicity Assay Using xCELLigence System

Real-time cytotoxicity was assessed using the xCELLigence RTCA SP system (Agilent Technologies, Santa Clara, CA, USA) with E-Plate 96. WI-38 fibroblasts (2 × 10^4^ cells per well) were seeded into E-Plate 96 and allowed to settle at room temperature for 30 min before continuous monitoring of the cell index (CI) at 37 °C in 5% CO_2_. After 24 h, once the CI had stabilized, effector cells were added as indicated for each experiment. For baseline functional assessment, FAP-CAR-NK cells were added at the indicated E:T ratio, and target-only wells were included as controls (*n* = 3).

Recombinant human TGF-β1 (MedChemExpress (MCE), Monmouth Junction, NJ, USA, #HY-P7118, expressed in CHO cells) was reconstituted according to the manufacturer’s protocol. Briefly, the lyophilized protein was centrifuged and dissolved in sterile ddH_2_O to obtain a stock solution. For long-term storage, the stock was further diluted to a concentration of ≥100 μg/mL in a buffer containing 5% trehalose as a carrier protein, aliquoted into single-use fractions, and stored at −80 °C. On the day of each experiment, one aliquot was thawed and freshly diluted to a final concentration of 5 ng/mL in complete DMEM (supplemented with 10% FBS). MRC-5 cells were serum-starved in DMEM containing 1% FBS for 6 h and then stimulated with 5 ng/mL TGF-β1 in complete DMEM for 48 h. After upregulation of FAP and α-SMA was confirmed by Western blot, RTCA killing assays were performed. The procedures were the same as described above. E:T ratios were set at 2:1, 1:1, and 1:2, and target cell-only wells were included as controls.

### 4.7. Western Blot

TGF-β-treated MRC-5 cells were lysed in RIPA (Servicebio, Wuhan, Hubei, China, #G2002-30ML) buffer supplemented with protease inhibitors (Servicebio, #G2006-250UL). Total protein was collected after centrifugation and quantified using a BCA assay (Servicebio, G2026-200T). Protein samples were mixed with 5× SDS-PAGE loading buffer at a 4:1 ratio, denatured at 70 °C for 15 min, separated by SDS-PAGE, and transferred onto 0.45 μm PVDF membranes (Servicebio, #GB6047-50-0.45). In this experiment, 5 μL of sample was loaded per lane. Membranes were blocked for 30 min at room temperature and incubated overnight at 4 °C with primary antibodies against FAP (Proteintech Group(Wuhan), Wuhan, Hubei, China, #11779-1-AP, 1:1000), α-SMA (Servicebio, #GB111364, 1:1000), and GAPDH (Servicebio, #GB15004, 1:1000), followed by HRP-conjugated goat anti-rabbit secondary antibody (Servicebio, #GB23303, 1:3000). Signals were detected using ECL reagent (Servicebio, #G2161-200ML) and quantified by densitometric analysis.

### 4.8. Carboxyfluorescein Succinimide Ester Proliferation Assay

Untransduced NK cells and FAP-CAR-NK cells were labeled with carboxyfluorescein succinimidyl ester (CFSE; MCE, #HY-D0938) according to the manufacturer’s instructions. A portion of the labeled cells was analyzed immediately after staining (0 h) to establish baseline fluorescence. The remaining cells were co-cultured with K562-FAP-Luc target cells for 48 h and then stained with anti-CD56 antibody for flow-cytometric analysis. Proliferative activity was evaluated based on CFSE fluorescence dilution within the CD56^+^ effector-cell population.

### 4.9. Degranulation and Exhaustion Phenotype Analysis

For degranulation analysis, untransduced NK cells and FAP-CAR-NK cells were co-cultured with K562-FAP-Luc target cells at an E:T ratio of 1:2 for 4 h. After incubation, cells were harvested and stained with antibodies against CD3, CD56, and CD107a. CD107a expression within the CD3^−^CD56^+^ NK-cell population was analyzed by flow cytometry to assess degranulation activity.

For exhaustion phenotype analysis, untransduced NK cells and FAP-CAR-NK cells were co-cultured with target cells at an E:T ratio of 2:1 for 24 h at 37 °C. Following incubation, cells were collected and stained with antibodies against CD56 and the exhaustion-associated markers PD-1, TIM-3, and LAG-3. Flow-cytometric analysis was performed on the gated CD56^+^ cell population to evaluate phenotypic changes after a single prolonged antigen exposure. No exogenous cytokines were supplemented during either co-culture condition.

### 4.10. Flow Cytometry Analysis

Flow cytometry was used to assess CAR expression, phenotype, and functional marker expression in effector-cell populations. For surface staining, approximately 5 × 10^5^ cells were collected, washed with cold PBS, and resuspended in staining buffer (PBS containing 2% FBS). Cells were incubated with pre-titrated fluorescently labeled antibodies for 30 min at 4 °C in the dark, washed, and resuspended in PBS for acquisition. All samples were analyzed on a CytoFLEX flow cytometer (Beckman Coulter, Brea, CA, USA), and data were processed using FlowJo software (version 10.8.1, BD Biosciences, Franklin Lakes, NJ, USA). Initial gating was performed using FSC-A and SSC-A parameters, followed by doublet exclusion. Marker expression was then analyzed within the relevant gated cell populations as specified in each assay. Antibody details are provided in Supplementary Table S1.

### 4.11. Lung Organoids Culture and Preparation

Human lung organoids obtained from a commercial biospecimen repository were maintained and expanded in a Matrigel-based culture system. Before subsequent experiments, organoids were recovered from Matrigel and processed into small multicellular clusters. In parallel, FAP-positive fibroblasts were cultured under hypoxic conditions as adherent cells and collected on the day of encapsulation. To construct the three-dimensional co-culture model, organoid clusters and fibroblasts were separately resuspended in Matrigel and loaded into a droplet-based microfluidic chip system (Chuangxin International (Accurate International), Guangzhou, Guangdong, China), with organoids introduced through the central channel and fibroblasts through the lateral channels. The resulting microdroplets were collected in a mixed culture medium and cultured for 3 days to establish the organoid–fibroblast co-culture model.

A pulmonary fibrosis-like state was induced by combined stimulation with TGF-β at 25 ng/mL and LPS at 500 ng/mL. Because this part of the model construction procedure was performed by a third-party technical platform under a confidentiality agreement, some proprietary operational parameters cannot be disclosed. To ensure methodological transparency, this study provides the non-proprietary information directly relevant to data interpretation to the greatest extent possible, including experimental grouping, sampling time points, co-culture ratios, and major endpoints. Specifically, the experimental groups included Con, PC, PC + NK, PC + CAR-NK, and PC + pirfenidone. Samples were collected 48 h after model establishment, and the co-culture ratio of NK/CAR-NK cells to lung organoids was 5:1–10:1.

### 4.12. Co-Culture and Pharmacological Interventions

After establishment of the fibrotic organoid co-culture model, experimental groups were assigned as Con, PC, PC + NK, PC + CAR-NK, and PC + PFD. For cell-based intervention groups, untransduced NK cells or FAP-CAR-NK cells were added to fibrotic organoids at the indicated effector-to-organoid ratios. Pirfenidone was included as a pharmacological antifibrotic control at 50 μM (MCE, #HY-B0673). Samples were collected 48 h after treatment for downstream analyses.

### 4.13. Detection and Evaluation

To dynamically monitor the behavior of different cellular components in the fibrotic organoid co-culture system, bright-field and multichannel fluorescence time-series imaging was performed using a BioTek Cytation 5 multimode imaging system (Agilent BioTek, Winooski, VT, USA). To distinguish different cell populations, CAF cells were pre-labeled by retroviral transduction with eGFP; untransduced NK cells and FAP-CAR-NK cells were stained with the red fluorescent probe CellTracker Deep Red (Thermo Fisher Scientific, Waltham, MA, USA, #A66460); and human lung PDOs were stained with the blue fluorescent probe CellTracker Blue CMAC (Thermo Fisher Scientific, #C2110). After establishment of the co-culture system, images were acquired every 6 h under identical imaging settings until the 48 h endpoint to record dynamic changes in the red, green, and blue fluorescence signals, thereby allowing evaluation of the quantitative changes and spatial distribution patterns of different cellular components during co-culture. All images were acquired using uniform exposure time, gain, objective magnification, and field-selection settings to minimize systematic variation between time points.

According to the experimental design, co-culture imaging was performed in low-attachment 96-well plates with duplicate wells. For pathological analysis, treated organoid samples were fixed, embedded, sectioned, and subjected to H&E staining and FAP immunohistochemistry (Abcam, Cambridge, UK, #ab207178, 1:100). H&E staining was mainly used to evaluate organoid structural integrity, the compactness of spheroid-like structures, and changes in peripheral cellular distribution, whereas FAP-IHC was mainly used to assess changes in the FAP-positive area. All images were acquired in the same batch under consistent parameters and were quantified according to a unified analysis standard. For molecular analysis, total RNA was extracted from organoids, and the mRNA expression levels of SP-C, FAP, COL1A1, and ACTA2 (α-SMA) were determined by RT-qPCR.

### 4.14. Acute Bleomycin-Induced Lung Injury Model with Irradiation-Based Preconditioning

All animal procedures were approved by the Institutional Animal Care and Use Committee (IACUC) of Shouzheng Hongyao Biotechnology Co., Ltd. (Wuhan, China; Approval No. 2025091601) and were conducted in accordance with institutional and national guidelines. Six-week-old male C57BL/6J mice were housed under specific pathogen-free conditions and acclimated for 1 week before experimentation.

Bleomycin-induced lung injury was established by trans-oropharyngeal laryngeal administration of bleomycin (50 μL of 1 mg/mL solution; 50 μg per 20 g body weight). On day 7 after bleomycin administration, mice received fractionated total body irradiation (TBI) consisting of 5.5 Gy, followed 4 h later by 4.5 Gy (0.75 Gy/min) to facilitate short-term persistence of infused human effector cells. Peripheral blood was collected 24 h after TBI to assess bone marrow depletion by flow-cytometric quantification of CD45^+^ leukocytes after red blood cell lysis.

At 72 h after TBI, mice were randomly assigned to receive tail vein infusion of PBS, untransduced NK cells, or FAP-CAR-NK cells (5 × 10^6^ cells in 200 μL PBS). At the time of infusion, mice received a single intraperitoneal injection of recombinant human IL-15 (3 μg/kg; GenScript, #Z03308) to support short-term effector-cell survival. For the primary acute-feasibility cohort, mice were euthanized 72 h after infusion for lung tissue collection. The left lung lobe was fixed in 4% paraformaldehyde for histological analysis, and the remaining lung tissue was collected for downstream assays. In a separate cohort, lung tissue was collected 24 h after infusion to assess acute pulmonary homing/engraftment of human cells by flow cytometry.

### 4.15. Histopathological Analysis

Fixed lung tissues were paraffin-embedded and sectioned at 4 μm thickness using a Leica RM2016 rotary microtome (Leica Microsystems, Wetzlar, Germany). Deparaffinized sections were subjected to hematoxylin–eosin (H&E) staining and Masson’s trichrome staining using commercial kits (Pinuofei, Wuhan, Hubei, China, #S191003 and #S191006, respectively) according to the manufacturers’ protocols. For FAP immunohistochemistry (IHC), sections were subjected to heat-induced antigen retrieval in sodium citrate buffer (pH 6.0), followed by blocking of endogenous peroxidase activity and nonspecific binding. Sections were incubated overnight at 4 °C with anti-FAP antibody (Abcam, #ab207178; 1:100), followed by HRP-conjugated secondary antibody (Abcam, #ab6721; 1:500), DAB visualization, and hematoxylin counterstaining. Images were acquired using a Nikon Eclipse-Ci upright microscope (Nikon Instruments, Tokyo, Japan), and quantitative image analysis was performed as indicated for each assay.

Histological and IHC quantification were performed using a unified workflow. For each mouse, three non-overlapping fields were randomly selected from lung tissue sections and imaged under identical microscope settings. For Masson’s trichrome staining, the percentage of collagen-positive area relative to total tissue area was calculated using ImageJ (version 1.54r). For FAP-IHC, the proportion of DAB-positive area or the integrated optical density (IOD) was quantified after color deconvolution using a uniform threshold. The mean value from multiple fields for each animal was used as the final value for statistical analysis. Given the limited sample size in this experiment, all quantitative results were used primarily to describe directional changes consistent with the study hypothesis.

### 4.16. Statistical Analysis

Data are presented as mean ± SEM. Unless otherwise specified, *n* in the figures represents the number of independent biological replicates. For cell-based experiments, *n* generally refers to experiments performed using effector cells derived from different donors or from independent preparation batches; replicate wells within the same plate were treated as technical replicates, and their mean value was used as one biological replicate for statistical analysis. For organoid experiments, n refers to the number of independently established organoid co-culture samples; multiple fields within each sample were averaged and were not treated as independent n values. For animal experiments, *n* refers to the number of animals included in the analysis for each group. Two-group comparisons were analyzed using paired two-tailed Student’s *t*-test where appropriate. Comparisons among three or more groups were analyzed by one-way ANOVA followed by Tukey’s post hoc test. The exact number of biological replicates (n) and the statistical test used for each dataset are indicated in the corresponding figure legends. Because of the very small number of surviving animals in the acute in vivo cohort, animal histological and IHC data were analyzed descriptively and were not used for formal inferential efficacy testing. *p* values < 0.05 were considered statistically significant.

## Data Availability

The data presented in this study are available upon request from the corresponding author.

## Supplementary Materials

The following supporting information can be downloaded at: https://www.mdpi.com/article/10.3390/ijms27094128/s1. References [7,38,39] are cited in the Supplementary Materials.

## Abbreviations

The following abbreviations are used in this manuscript:

FAP	Fibroblast activation protein
PF	Pulmonary fibrosis
ECM	Extracellular Matrix
AT2	Alveolar Epithelial Type II Cells
SP-C	Surfactant Protein C
CAF	Cancer-Associated Fibroblast
ATCC	American Type Culture Collection
ScFv	Single-Chain Variable Fragment
CAR	Chimeric Antigen Receptor
α-SMA	Alpha-Smooth Muscle Actin
CFSE	Carboxyfluorescein Succinimidyl Ester
PDOs	Patient-Derived organoids
RTCA	Real-Time Cell Analysis
Luc	Luciferase
TBI	Total body irradiation
PFD	Pirfenidone
BLM	Bleomycin
