# Supplementary material for: Proof-of-Concept Evaluation of Primary Human FAP-CAR-NK Cells Targeting Activated Fibroblasts in Pulmonary Fibrosis
Source: Int J Mol Sci. 2026 May 5;27(9):4128. doi: 10.3390/ijms27094128 (PMC13164303; doi:10.3390/ijms27094128)
Supplement: Supplementary file 1 [file ijms-27-04128-s001.zip › Figure s1.pdf]

Figure S1. Validation of retroviral production and packaging system for FAP-CAR.

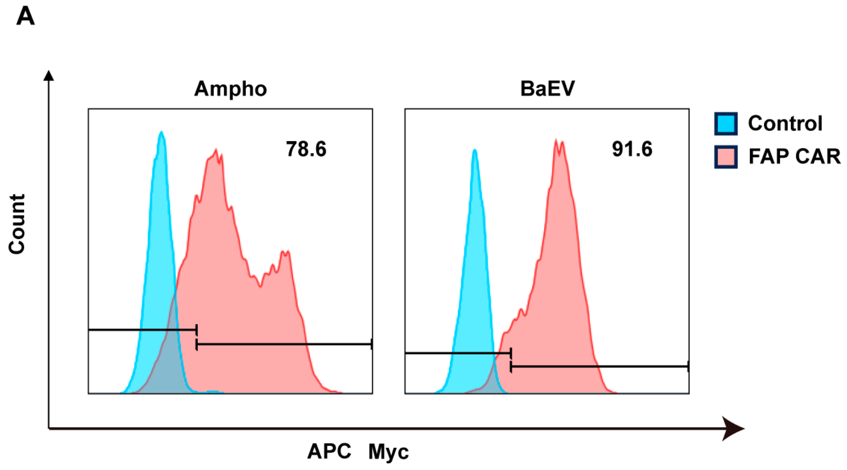

Figure S1 (A) Comparison of transduction efficiency between Ampho and BAEV packaging cell lines.
