# Supplementary material for: Proof-of-Concept Evaluation of Primary Human FAP-CAR-NK Cells Targeting Activated Fibroblasts in Pulmonary Fibrosis
Source: Int J Mol Sci. 2026 May 5;27(9):4128. doi: 10.3390/ijms27094128 (PMC13164303; doi:10.3390/ijms27094128)
Supplement: Supplementary file 1 [file ijms-27-04128-s001.zip › Figure s2.pdf]

Figure S2. Construction of K562-FAP-Luc target cells.

A

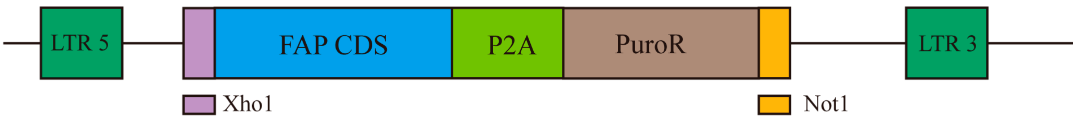

B

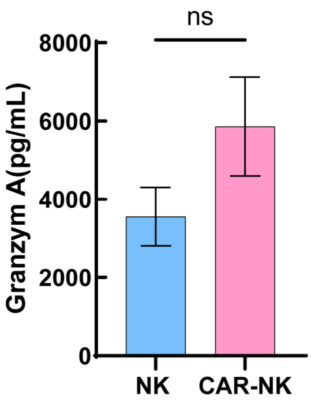

Figure S2 (A) Schematic representation of the FAP overexpression plasmid construct. (B) Supplementary cytokine profiling results assessed by cytometric bead array (CBA).
