# Supplementary material for: Proof-of-Concept Evaluation of Primary Human FAP-CAR-NK Cells Targeting Activated Fibroblasts in Pulmonary Fibrosis
Source: Int J Mol Sci. 2026 May 5;27(9):4128. doi: 10.3390/ijms27094128 (PMC13164303; doi:10.3390/ijms27094128)
Supplement: Supplementary file 1 [file ijms-27-04128-s001.zip › Figure s3.pdf]

Figure S3. Time-course imaging of fibrotic lung organoid co-cultures.

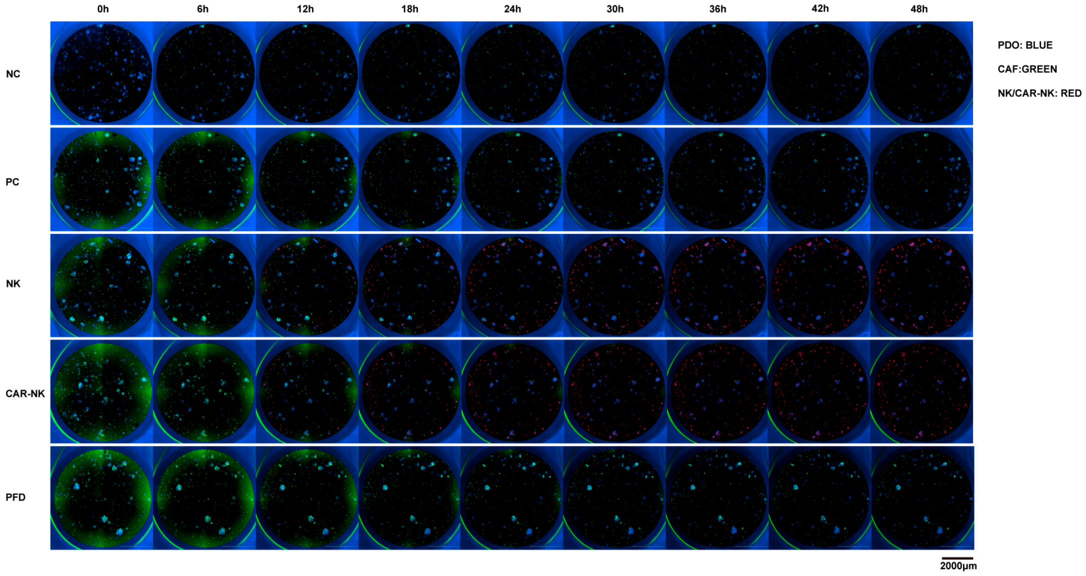

Figure S3 48-hour total cultivation for fluorescence time-lapse imaging. blue = lung organoids / CMAC, green = HFL-1 or FAP-positive fibroblasts / GFP, red = NK or CAR-NK / CellTracker Deep Red.
