# Supplementary material for: Proof-of-Concept Evaluation of Primary Human FAP-CAR-NK Cells Targeting Activated Fibroblasts in Pulmonary Fibrosis
Source: Int J Mol Sci. 2026 May 5;27(9):4128. doi: 10.3390/ijms27094128 (PMC13164303; doi:10.3390/ijms27094128)
Supplement: Supplementary file 1 [file ijms-27-04128-s001.zip › Table S1.pdf]

Table S1. Antibodies used in this study.

Name	Reactivity	Cat#	Company
APC anti-human CD3	Human	981012	Biolegend
PE/Cy7 anti-human CD56	Human	985912	Biolegend
Human C-Myc PE	Human	IC3696P	R&D systems
APC anti-C-Myc	Human	626809	Biolegend
Human FAP APC	Human	FAB3715A-025	R&D systems
PE/Cy7 anti-human CD107a	Human	328617	Biolegend
FITC anti-mouse CD45	Mouse	103108	Biolegend
APC anti-human CD45	Human	982304	Biolegend
