# Supplementary material for: Proof-of-Concept Evaluation of Primary Human FAP-CAR-NK Cells Targeting Activated Fibroblasts in Pulmonary Fibrosis
Source: Int J Mol Sci. 2026 May 5;27(9):4128. doi: 10.3390/ijms27094128 (PMC13164303; doi:10.3390/ijms27094128)
Supplement: Supplementary file 1 [file ijms-27-04128-s001.zip › Table S2.pdf]

Table S2. Primer sequences used for RT-qPCR.

Gene	Forward	sources
COL1A1-F	ATGTTCAGCTTTGTGGACCTC	[38]
COL1A1-R	CTGTACGCAGGTGATTGGTG	
α-SMA(ACTA2)-F	CTGTTGCAGCCATCCTTCAT	[38]
α-SMA(ACTA2)-R	TCATGATGCTGTTGTAGGTGG	
SFTPC(SP-C)-F	ATCCCCAGTCTTGAGGCTCT	[39]
SFTPC(SP-C)-R	CTTCCACTGACCCTGCTCAC	
FAP-F	CAAAGGCTGGAGCTAAGAATCC	[7]
FAP-R	ACTGCAAACATACTCGTTCATCA	
